# Does global positioning system-based navigation dependency make your sense of direction poor? A psychological assessment and eye-tracking study

**DOI:** 10.3389/fpsyg.2022.983019

**Published:** 2022-10-06

**Authors:** Wanling Yan, Jialing Li, Can Mi, Wei Wang, Zhengjia Xu, Wenjing Xiong, Longxing Tang, Siyu Wang, Yanzhang Li, Shuai Wang

**Affiliations:** ^1^School of Psychology, Chengdu Medical College, Chengdu, China; ^2^School of Clinical Medicine, Chengdu Medical College, Chengdu, China

**Keywords:** global positioning system, dependency, spatial cognition, sense of direction, eye-tracking tasks, correlation, impulsive, internet gaming

## Abstract

**Background:**

Global positioning system (GPS)-based navigation apps are very useful in our lives. However, whether and how the usage of these apps affects spatial cognition and the sense of direction is still unclear.

**Methods:**

A total of 108 individuals were recruited and completed the GPS dependence, internet gaming behavior, and impulsivity test using scales. The eye-tracking-based general mental rotation (MR) task and target finding (TF; require individuals to find a target specified in a 3D street map in a rotated version of top 2D view map) task were used to assess their spatial cognition and the sense of direction. The correlation was used to relate GPS navigation usage, spatial cognition ability, and impulsivity. Subgroup analyses stratifying by gaming hours of individuals (< 2 h or ≥ 2 h) or maps (countryside or city) in TF task were performed. The moderating and mediating effect analyses were conducted to verify these relationships.

**Results:**

The GPS dependency score was nominal positively correlated with fixations in the TF task in the entire cohort (*r* = 0.202, unadjusted *p* = 0.036); it was significant in city (*r* = 0.254, *p* = 0.008) and gaming time of < 2 h (*r* = 0.459, *p* = 0.001) subgroups. The high-score (upper 30%) group of GPS dependency had more fixations on the original target building in the training area and indicative building in the test area than the low-score (lower 30%) group. GPS dependency was not associated with the correct rate and reaction time in the TF task or any of the indicators in the MR task (*p* > 0.05). The GPS dependency mediated the indirect effect of impulsivity on the fixations on TF. The internet gaming time moderated the association between GPS dependency and fixations on TF.

**Conclusion:**

The dependency on GPS-based navigation apps was associated with impaired spatial cognition but may not significantly affect the sense of direction.

## Introduction

Global positioning system (GPS)-based navigation apps, which can acquaint individuals with an unfamiliar environment and guide them to correct destinations, have become a handy tool in our lives (Wang et al., 2018). According to the data obtained from iiMedia Research, as of the first quarter of 2019, the number of monthly active users of GPS-based navigation mobile apps, such as AutoNavi or Baidu map, has exceeded 300 million in China (Life and Travel Industry Research Center, 2019). The main reason why this type of app is so popular is that it minimizes the need for using our internal navigation system, reduces the cognitive requirements of navigation and the probability of mistakes, and further saves our energy in finding our target location (Dahmani and Bohbot, 2020). Further, some people, such as those with high impulsivity, have a higher perceived dependence on and actual use of mobile phones, in which the majority of active usage was due to the GPS-based navigation mobile apps (Billieux et al., 2007; Verdejo-Garcia et al., 2007; van Endert and Mohr, 2020). However, the long-term use of such apps might harm spatial map-building ability and further impair spatial cognition (Gramann et al., 2017).

Spatial cognition is concerned with acquiring, organizing, utilizing, and revising knowledge about spatial environments, related to how people talk about their environment, find their way in new surroundings or plan routes (Burgess, 2008). Spatial cognition is built by spatial memory and stimulus-response strategies to store and use information about landmarks and the routes between landmarks (Werner et al., 1997). Spatial memory is responsible for recording and recovering information about the surrounding environment, architecture, and orientation (Bohbot et al., 2004; Eichenbaum, 2017). The stimulus-response strategy is responsible for learning and remembering motor responses, such as turning (Squire and Zola, 1996; Schwabe et al., 2007; Dahmani and Bohbot, 2020). Whether the pathfinding event could be completed successfully is affected by the ability of spatial cognition, which includes the extent of use of the above strategies. The sense of direction, related to spatial awareness and spatial cognition, is the ability to know one’s location and perform wayfinding (Kozlowski and Bryant, 1977; Cornell et al., 2003). Spatial cognition could be simply measured by mental rotation (MR) tasks (Pietsch and Jansen, 2012). MR can be described as the brain moving objects to help understand what they are and where they belong. Some studies using this task showed that male individuals, long-time internet gaming participants, and sportspeople might have better spatial cognition (De Lisi and Cammarano, 1996; Feng et al., 2007; Spence and Feng, 2010; Jansen et al., 2012). However, the task is still far from the actual pathfinding event. Therefore, the development of a task with a virtual city with both landmarks and routes may be more meaningful in testing spatial cognition and the sense of direction.

When using a GPS-based navigation app, the brain might rarely invoke spatial cognition (Dahmani and Bohbot, 2020). Using a GPS involves the following step-by-step sensorimotor instructions (e.g., turn right at the next intersection, turn left in 500 m) (Dahmani and Bohbot, 2020). Previous studies using fMRI and a simulation in London (United Kingdom) revealed that the brain regions involved in spatial cognition had increased activity when entering a street during GPS navigation (Javadi et al., 2017). A survey of 50 regular drivers suggested that the habitual use of GPS impacted their spatial memory negatively during self-guided navigation (Dahmani and Bohbot, 2020). However, Yang’s study used the “Questionnaire on Spatial Strategies (Münzer et al., 2016)” to evaluate the environmental spatial cognition in 550 GPS-based navigation app users and showed that the frequency of mobile navigation usage did not significantly associate with the scores in this questionnaire, indicating that GPS using necessarily damage individuals’ environmental spatial cognition (Yang, 2020).

Judging from the above evidence, the conclusions about whether and how the usage of GPS-based navigation apps affects spatial cognition and the sense of direction are inconsistent and still vague. Further, simulated reality-based navigation tasks have not been developed and used in these studies. Hence, there also have a gap between the conclusions and the reality of pathfinding events. In this study, we hypothesized that long-term usage or dependency on GPS-based navigation apps might decrease spatial cognitive ability and the sense of direction. An eye-tracking technology-based target finding (TF; require individuals to find a target specified in a 3D street map in a rotated version of top 2D view map) task was developed to explore the changes in spatial memory and stimulus-response strategies in individuals through a simulated pathfinding event in a 3D view of a city or countryside, which was closer to the actual scene. Further, we also hypothesized that impulsivity might indirectly affect spatial cognition *via* the usage of GPS-based navigation apps, and internet gaming behavior and gender might affect spatial cognition.

## Materials and methods

### Participants

College students between 18 and 25 were recruited to participate in this cross-sectional study. Participants were required to be right-handed and to report having no history of drug or alcohol abuse, general neurological or psychiatric disorders, or behavioral addiction, such as internet gaming disorder or social media disorder. Additionally, individuals who cannot walk independently or have a history of head trauma followed by a loss of consciousness were excluded. No requirements for GPS use were limited to recruitment.

The studies involving human participants were reviewed and approved by the Medical Ethics Committee of Chengdu Medical College. The participants provided their written informed consent to participate in this study.

A total of 108 individuals were recruited and had finished all the evaluations in the study. The mean age of the cohort was 19.85 years old. 67.6% were male.

### Impulsivity assessment

Impulsivity might relate to the actual use of GPS navigation and further impact spatial cognition. The Chinese version of the Barratt impulsiveness scale (BIS) in the 11th (Yang et al., 2007) with a Cronbach’s alpha of 0.76 was used to measure the impulsivity of the participants. The BIS-11 scale has a 4-point Likert scale with 26 items divided into the three dimensions of attention, non-planning, and motor impulsiveness. The scores for questions 1, 6, 7, 8, 9, 11, 12, 14, 18, 25, and 26 are reversed in this scale. The higher the scale score, the more impulsive the participants.

### Gaming behavior assessment

Considering that studies suggested that internet gaming might affect spatial cognition (Spence and Feng, 2010), information on internet gaming behavior (daily gaming time and gaming type) was also collected. Individuals with an average daily gaming time of ≥ 2 h in the past 3 months were classified as high gaming hours daily players (HGHD) (Jaruratanasirikul et al., 2009). Hence, in this study, the participants were divided into HGHD (daily gaming time of ≥ 2 h; *n* = 50) and non-HGHD (daily gaming time of < 2 h, including the no-gaming individuals; *n* = 58). The gaming type was recorded in HGHD and divided into role-playing (*n* = 29) and others (*n* = 21).

### Global positioning system dependency assessment

The Chinese translation of the GPS dependence scale (Dahmani and Bohbot, 2020) was used to assess the GPS experience of the participants, which mainly assesses the individual’s actual use of GPS, especially walking or driving to a new or known target location, by using the GPS-based navigation apps in mobile phone. The GPS dependence scale has a 5-point Likert scale with 13 items and measures the extent to which people feel dependent on their GPS. Participants are asked to consider the past month as they answer the questions. Each response corresponds to a number, and the total score is calculated based on the sum of the individual responses. The scores for questions 3, 6, and 12 are reversed on this scale. The higher the total score on the scales is, the more dependent the participant was on GPS-based navigation. The original English version is detailed in Dahmani’s study (Dahmani and Bohbot, 2020). Our pilot study with 122 Chinese college students was used to validate the Chinese translation scale. The Cronbach’s alpha and split-half reliability of this scale were 0.76 and 0.74, respectively. The retest reliability in 30 individuals was 0.70.

### Spatial cognition ability assessments

**FIGURE 1:**
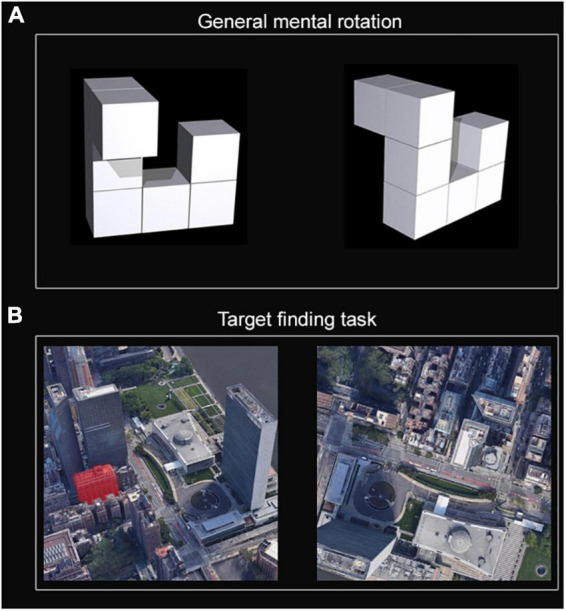
Eye-tracking tasks. **(A)** General mental rotation (MR). **(B)** Target finding (TF) task.

The MR and TF tasks were used to evaluate the participants’ spatial cognition ability and sense of direction based on the eye-tracking system. The MR task was relatively simple, and subjects needed to retrieve and memorize little information (the number of blocks and these relative positions). The TF task was closer to the realistic pathfinding events. The subjects needed to remember the surrounding environment, landmarks of the target, and their relative position and direction. Further, the TF task also requires calling on the subject’s mental rotation ability. The design of the MR task (Figure 1A) is detailed in our previous study (Wang et al., 2022).

The present MR task consisted of 20 unique block pairs resulting in a 50% “same” and 50% “different” result (Wang et al., 2022). The stimuli were presented pairwise in two different angular disparities of 45° and 90°, and each figure had a dimension of 400 × 400 pixels (Wang et al., 2022). A fixation stimulus (a white cross) appeared at the center of the screen at the start of each trial. The neutral fixation stimulus changed to a task trial when the participants maintained central fixation for 1,600 ms. Within 10,000 ms in each trial, the participants must determine the final judgment and press “Y” for “same” and “N” for “different.” When a trial was completed, a blank appeared for 500 ms (Wang et al., 2022).

The TF task (Figure 1B) was developed based on a simulated navigation task in a 3D street without GPS. In this task, participants needed to invoke their brain’s spatial memory and stimulus-response strategies to remember the characteristics of the target and its surroundings. Two maps on a black background will be presented in each test in the TF task. The left map with an original target building (marked in red) in a 3D street view was defined as a training area, which will display 15,000 ms at the beginning of a test. Participants were required quickly remember this target’s characteristics, relative position, and surroundings, including landmarks and routes. The right map was the top 2D view (without a red marker) of the 3D map on the left after being rotated by a certain angle and defined as the test area. It will display for 30,000 ms after the 1,500 ms interval of the left map disappears. The correct answer was defined as the participant finding the target and kept fixating on the building in the right testing area for 3,000 ms. The answering was limited to being completed within 30,000 ms. Otherwise, it will be judged as a failure. After an introduction and pre-test, there were ten formal tests (maps: 4 for the countryside and 6 for the city) in the TF task. A 5,000 ms rest period after each test was set in the TF task. A pilot study was used to validate this task and showed a strong correlation (*r* = 0.628, *p* < 0.001) between its correct rate and the Santa Barbara sense of direction scale (Hegarty et al., 2002). Further, the indicators in TF tasks, which could reflect spatial memory and search, were strongly associated with indicators in MR task in the present study. These implied that the TF task had high applicability for populations.

The EyeLink 1000 system (SR Research Ltd., Canada) was used for performing these tasks. Dominant eye movements were recorded at a sampling rate of 1,000 Hz. With the Experiment Builder software (SR Research Ltd., Canada), the two tasks were presented on a computer monitor (1,024 × 768 resolution, 60 Hz refresh rate) at a distance of 50 cm from the participant’s eyes. Parameters such as correct rate, reaction time (average reaction time in the tests which were correctly answered), and fixation (average number of fixation count in the tests which were correctly answered) were recorded in the two tasks. The correct rate was calculated as the ratio of correct answers to the total number of trials. Reaction time was defined as the time from the start to the correct answer. A fixation that we recognize in a sample can start at any time between the previous target (when we saw there was no saccade) and the current target. The number of fixation count reflects the efficiency of target search and memory. Higher correct rates, shorter reaction times, or fewer fixations indicate better spatial cognition ability and sense of direction.

### Data and statistical analyses

The eye-tracking task data were extracted using DataViewer (SR Research Ltd., Canada). The data on impulsivity, internet gaming behavior, and GPS-based navigation dependency assessments were manually extracted from the original questionnaires. The primary outcome was set as the association between GPS dependency scores and indicators in spatial cognition ability, which was analyzed using Pearson or Spearman’s correlations in SPSS 22.0 (IBM Co., United States). Subgroup analyses were also conducted after the cohort was stratified by the map in the TF task (city or countryside), internet gaming behavior (HGHD or non-HGHD; role-playing or others), and gender (male or female). Heatmaps, only allowing comparisons between groups, were used to detect further the differences in the eye-tracking characteristics between the low-score GPS dependency group (lower 30%) and high-score group (upper 30%) by DataViewer. The upper and lower rule is commonly used in item analysis in psychology based on Kelley’s derivation (Kelley, 1939). Bonferroni corrections were applied to avoid a type I error in the multiple tests. Generally, the statistical analyses were two-tailed, and the level of statistical significance was adjusted to *p* < 0.05 (unadjusted *p* < 0.0083). The secondary outcomes were set as the proving of the model: impulsivity indirectly affects the fixations in TF task *via* GPS dependency, and internet gaming time moderates the effects of the usage of GPS-based navigation apps on the fixations in TF task. The moderating and mediating effect analyses were conducted by SPSS 22.0.

## Results

### Internet gaming behavior and impulsivity of the cohort

46.3% of participants (*n* = 50) had more than two gaming hours daily, considered HGHD (Table 1), 29 of whom were role-playing gamers. These general characteristics are consistent with the internet gaming population in China. Further, BIS’s total, attention, motor, and non-planning scores were 58.11, 13.79, 18.97, and 25.35 and followed a normal distribution (Table 1).

**TABLE 1:** The characteristics of the cohort.

Indicators	Values
Age (years, mean ± sd)	19.85 ± 1.34
Gender (Male/Female, *n*)	73/35
Gaming hours daily > 2 h (Yes/No, *n*)	50/58
Gaming type (Role-playing/Others, *n*)	29/21
**Barratt impulsivity scale**	
Total score (mean, range)	58.11, 43–76
Attention (mean, range)	13.79, 7–21
Motor (mean, range)	18.97,13–29
Non-planning (mean, range)	25.35, 13–37

### Global positioning system dependency of the cohort

**FIGURE 2:**
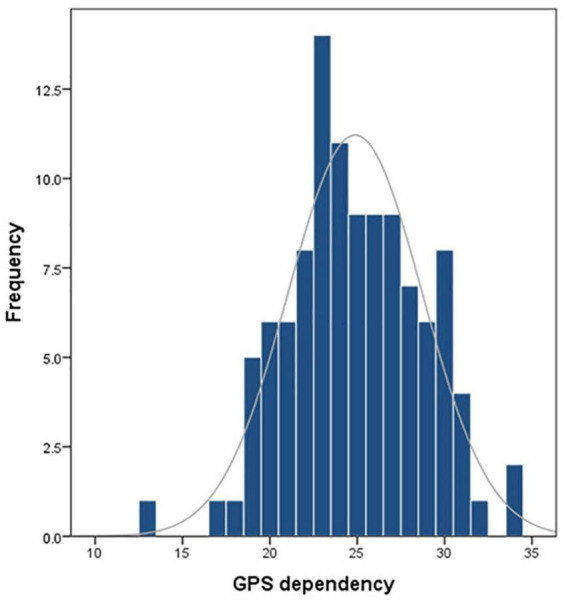
Distribution of GPS dependency score in the cohort.

The GPS dependency scales were used to evaluate the GPS-based navigation dependency in 108 individuals. The median score of this cohort in terms of GPS dependency was 25. As shown in Figure 2, the GPS dependency score in this cohort follows a normal distribution. There have weakly associations between GPS dependency score and BIS scores (total: *r* = 0.217, *p* = 0.024; attention: *r* = 0.265, *p* = 0.006). Further, the reaction time and fixations in both MR and TF tasks follow a normal distribution. Hence, the Pearson correlation coefficient was used in the analyses for these indicators. Spearman’s correlation was used in the correlations between GPS dependency and correct rate (in both MR and TF), which does not follow the normal distribution.

### Correlation between global positioning system dependency and spatial cognition ability

**FIGURE 3:**
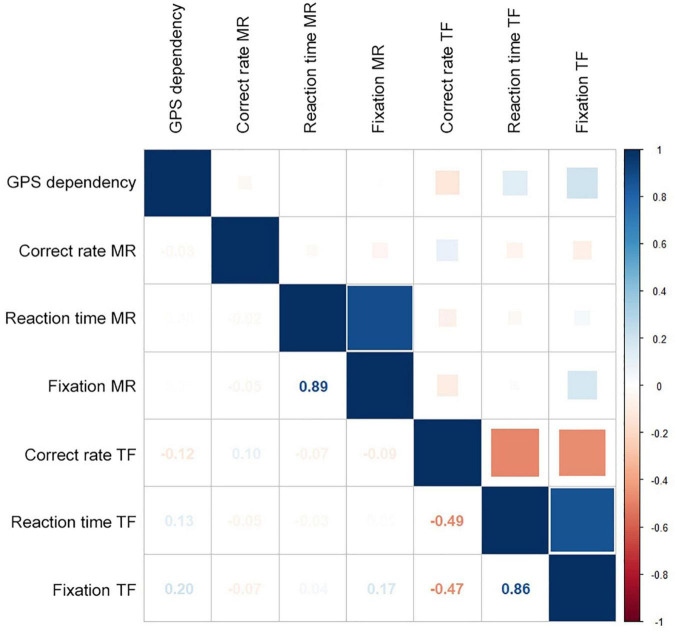
Correlation between GPS dependency score and indicators in eye-tracking tasks. Blue represents the positive correlation, and red represents the negative correlation. The numbers in the grids represent the correlation coefficient. MR represents mental rotation task. TF represents target finding task.

**FIGURE 4:**
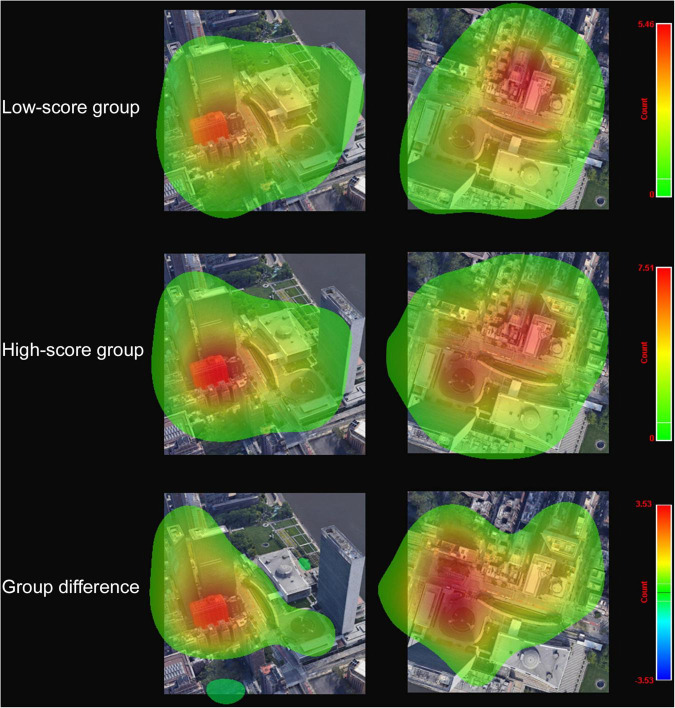
Eye-tracking heatmap of fixation count. The redder the color, the higher the number of fixation count when performing target finding task.

Two eye-tracking-based tasks were used to evaluate the spatial cognition ability of 108 individuals. The correct rate of this cohort was 78.30% and 70.90% in the MR and TF tasks (Table 2). The mean reaction time and fixations in these tasks were also detailed in Table 2. In the correlation analysis in Figure 3, only the GPS dependency score was nominal positively correlated with fixations in the TF task. However, it was not significant after Bonferroni correction (*r* = 0.202, unadjusted *p* = 0.036 > 0.0083). No significant correlation was found between GPS dependency and correct rate and reaction time in the TF task or all indicators in the MR task (unadjusted *p* > 0.05). Eye-tracking heatmaps were used to detect further the differences in the fixation characteristics between the low-score GPS dependency group (lower 30%) and the high-score group (upper 30%). As shown in Figure 4, the high-score group seemed to have more fixations on the original target building in the training area and indicated building in the test area than the low-score group.

**TABLE 2:** The spatial cognition ability of the cohort based on eye-tracking study.

Indicators	Mean value (SD)	Range
**General mental rotation**		
Correct rate (%)	78.30 (16.83)	10.00–100.00
Reaction time (s)	4.81 (1.43)	1.99–8.48
Fixations (times)	18.07 (4.82)	8.50–32.20
**Target finding task**		
Correct rate (%)	70.90 (24.55)	10.00–100.00
Reaction time (s)	14.94 (4.21)	6.82–27.98
Fixations (times)	39.52 (12.92)	12.63–93.00

**FIGURE 5:**
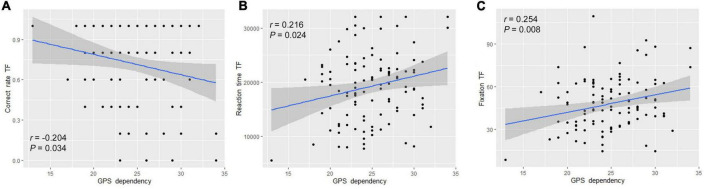
Correlation between GPS dependency and indicators in eye-tracking TF task in the city. **(A)** Correct rate; **(B)** reaction time; **(C)** fixation. TF represents target finding task.

**FIGURE 6:**
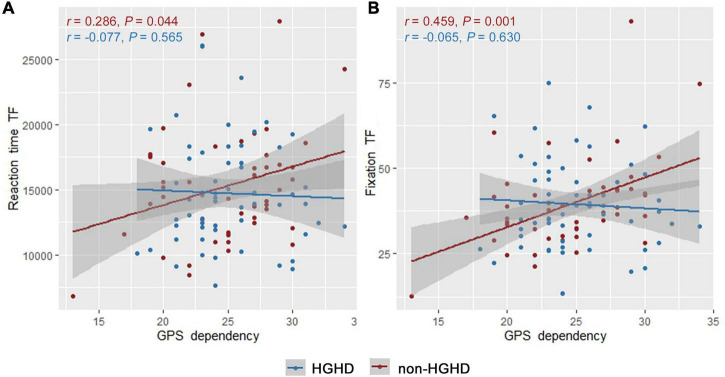
Correlation between GPS dependency and indicators in eye-tracking TF task in subgroup stratified by Internet gaming behavior. **(A)** Reaction time; **(B)** fixation. TF represents target finding task. HGHD represents high gaming hours daily players.

Subgroup analyses were then used to detect further the correlations between GPS dependency score and indicator (correct rate, reaction time, and fixation) in the TF task. In the virtual city (*n* = 108) but not the countryside, the GPS dependency score was significantly and weakly correlated with the fixations (*r* = 0.254, unadjusted *p* = 0.008 < 0.0083) (Figure 5). The subgroup was also stratified by daily gaming time, as studies suggested that internet gaming might affect spatial cognition (Spence and Feng, 2010). In HGHD individuals (*n* = 50), no significant correlation was found between GPS dependency score and indicator (correct rate, reaction time, and fixation) in the TF task (unadjusted *p* > 0.05, Figure 6). However, in non-HGHD individuals (*n* = 58), the GPS dependency score was significantly positively correlated with fixations (*r* = 0.459, unadjusted *p* = 0.001 < 0.0083) (Figure 6). However, when the subgroup was stratified by gender or gaming type, no significant correlation was found between GPS dependency and indicator (correct rate, reaction time, and fixation) in the TF task (unadjusted *p* > 0.05).

### Moderating and mediating effect analysis

**FIGURE 7:**
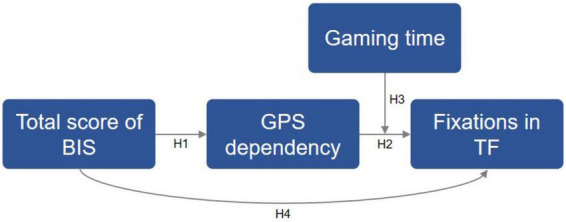
Paths in moderating and mediating effect analysis. H1, mediating model, a high level of impulsivity affects the GPS dependency negatively. H2, mediating model, higher GPS dependency may have more fixations in TF task. H3, moderating model, the association between GPS dependency and performance in TF task was significantly moderated by gaming time. H4, mediating model, impulsivity was negatively related to fixations in TF task. TF represents target finding task.

Correlation results in a cross-section study cannot prove a causal relationship between factors. Based on the correlation results and evidence above, we built one model with four hypothesizes (Figure 7): 1) individual with a high level of impulsivity may have a high dependency on GPS-based navigation (H1); 2) individual with higher GPS dependency may make more fixations in the TF task (H2); 3) the association between GPS dependency and fixations in TF task was significantly moderated by gaming time (H3); 4) impulsivity was negatively related to fixations in TF task (H4). The validation results (Table 3) indicated that the total score of BIS has an indirect but not direct effect on the fixations in TF task *via* GPS dependency (H1: *p* = 0.024; H2: *p* = 0.035; H4: *p* = 0.725). Furthermore, the associations of GPS dependency with the fixations in TF were moderated by internet gaming time (H3: *p* = 0.009).

**TABLE 3:** Hypothesis validation results.

Hypotheses (Path)	Estimation (95% CI)	*p*	Relationship
**Mediating effect**			
H1: Total score of BIS → GPS dependency	0.123 (0.016–0.229)	0.024	Supported
H2: GPS dependency → Fixation count of TF	0.705 (0.052–1.357)	0.035	Supported
H4: Total score of BIS → Fixation count of TF	–0.066 (–0.435–0.304)	0.725	No
**Moderating effect**			
H3: Gaming behavior for fixation count of TF	–3.221 (–5.618–0.824)	0.009	Supported

## Discussion

Spatial cognition ability, especially spatial memory, was considered impaired in long-term users of GPS-based navigation when finding destinations (Dahmani and Bohbot, 2020). In the present study, it appears that participants with different degrees of GPS-based navigation dependency did not have significant differences in the accuracy and reaction time of performing the MR or TF tasks. However, we mainly found that the participants with a higher score of GPS dependency had more fixations on the original target or indicative building when performing the city-related TF task, suggesting that GPS-based navigation dependency was associated with the decreased ability in the efficiency of spatial target search and memory when performing the pathfinding event. Furthermore, since the correct rate in the execution of TF and MR tasks of the high-dependent cohort did not decrease significantly, the sense of direction did not change significantly. In addition, the total score of BIS indirectly affects the fixations in the TF task *via* GPS dependency. The association of GPS dependency with the fixations in the TF task was moderated by internet gaming time.

Spatial memory has representations within working, short-term memory, and long-term memory. Short-term spatial memory could be described as a system that allows one to temporarily store and manage information that is necessary to complete complex cognitive tasks (Johnson, 2010). Furthermore, short-term spatial memory is a cognitive process that enables a person to remember different locations and spatial relations between objects (Johnson, 2010; Banta Lavenex et al., 2015). For instance, one can remember where an object is to another, allowing someone to navigate a familiar city (Berthoz and Viaud-Delmon, 2002; Johnson, 2010). Short-term spatial memory relies mainly on the dorsal hippocampus and prefrontal cortex (Seamans et al., 1998; Izaki et al., 2008). Patients with diseases of damage to the two brain regions, such as schizophrenia, were thought to have impaired short-term spatial memory and showed reduced ability in spatial cognition and sense of direction after hippocampal resection (Abrahams et al., 1997; Spieker et al., 2012). The present study’s tasks mainly assessed the participants’ short-term spatial memory. Individuals who depend highly on GPS needed a greater number of times and energy to observe and remember the target and surrounding environment and showed a poor ability to remember the features of a destination and surrounding environment. This finding is consistent with a previous study, which revealed that GPS users traveled more slowly and rated wayfinding tasks more difficult than direct-experience participants (Ishikawa et al., 2008). Hence, we speculated that this population might have an impairment of short-term spatial memory. However, whether this population has abnormal structure or function in the dorsal hippocampus and prefrontal cortex is still unclear, even though the study has shown that these two brain regions are overactivated during GPS navigation tasks (Javadi et al., 2017).

Internet gaming, especially role-playing games, is proven to improve sensory, perceptual, and attentional abilities that are important for many tasks in spatial cognition (Subrahmanyam and Greenfield, 1994; Spence and Feng, 2010). An interventional study on 47 children showed that participants whose initial mental rotation performance was low improved after playing computer games that entailed mental rotation skills (De Lisi and Wolford, 2002). Further, expert gamers could better track objects that move at incredible speeds and detect changes in objects stored in visual short-term spatial memory than non-gamers (Boot et al., 2008). These effects may be due to the increased gray matter in the hippocampus and the functional connectivity in the entorhinal cortex after having internet gaming experiences (West et al., 2018). The increase in spatial memory caused by internet gaming may offset the decrease in this ability because of GPS-based navigation. Therefore, this may explain why the correlations between GPS dependency or reliance and indicators in eye-tracking-based tasks in the present study were not significant in the HGHD but rather significant in the non-HGHD population.

The underlying reasons why people increasingly prefer to use GPS-based navigation are still vague. In this study, we further analyzed the association between GPS-based navigation dependency and impulsivity and attempted to detect the underlying reasons. The results suggested that GPS dependency was mainly associated with the impulsivity of the participant. Impulsivity is both a facet of personality and a major component of various disorders, including substance use disorders (Lane et al., 2003), bipolar disorder (Henry et al., 2001), and borderline personality disorder (Shorub et al., 2022). Acquired brain injury and neurodegenerative diseases were noted based on abnormal impulsivity patterns (Migliaccio et al., 2020). Impulsivity relates to perceived dependence on and actual use of mobile phones (Billieux et al., 2007). Neurobiological findings suggest that specific brain regions, such as part of the prefrontal cortex, are involved in impulsive behavior (Kim and Lee, 2011). Therefore, the correlation between impulsivity and GPS-based navigation dependence might be due to the joint influence of brain regions, such as the prefrontal cortex, which is dominated by impulse control and spatial memory. The final moderating and mediating effect analyses suggested that the high impulsivity of individuals makes them more dependent on GPS for travel, which reduces short-term spatial memory. In addition, gaming behavior moderates this effect, implying that individuals who are regularly trained through internet gaming have fewer adverse effects than non-gamers.

Several limitations of the present study should be considered. Although correlation analysis, mediation model, and other methods were used to explore the relationships among impulsivity, GPS-based navigation dependence, and spatial cognition, the conclusions still cannot explain a causal correlation because of the cross-sectional design of the present study. In addition, although we used 3D street maps to perform the task, the gap between the results and the real-world wayfinding event also existed, thereby making the results biased. In the future, we will further confirm our conclusions by performing real-world tasks in a longitudinal cohort.

## Conclusion

Over-dependence on GPS-based navigation apps was associated with the impairment of individuals’ short-term spatial memory in spatial cognition, thereby causing them to spend more time and energy observing and remembering the details in city wayfinding tasks. However, it may not significantly affect the sense of direction. The degree of reported impulsivity may influence the development of this dependence. Internet gaming experience may mitigate this adverse effect. The study may provide key evidence supporting the rational use of such apps and the use of online games to exercise spatial ability.

## Data availability statement

The original contributions presented in this study are included in the article/supplementary material, further inquiries can be directed to the corresponding author/s.

## Ethics statement

The studies involving human participants were reviewed and approved by Chengdu Medical College. The patients/participants provided their written informed consent to participate in this study.

## Author contributions

All authors contributed to the data analysis, drafting, and revision of this manuscript agreed on the journal to which the article will be submitted, gave final approval of the version to be published, and agreed to be accountable for all aspects of the work.
